# Aberrantly expressed messenger RNAs and long noncoding RNAs in degenerative nucleus pulposus cells co-cultured with adipose-derived mesenchymal stem cells

**DOI:** 10.1186/s13075-018-1677-x

**Published:** 2018-08-16

**Authors:** Zhihua Han, Jiandong Wang, Liang Gao, Qiugen Wang, Jianhong Wu

**Affiliations:** 1 Orthopedic Traumatology, Shanghai Trauma and Emergency Center, Shanghai General Hospital, School of Medicine, Shanghai Jiaotong University, Xin Songjiang Road 650, Shanghai, 201620 China; 2 Department of Orthopedics, The First Affiliated Hospital of Anhui Medical University, Hefei, China; 3 Experimental Trauma & Orthopedics, Frankfurt Initiative for Regenerative Medicine, J.W. Goethe University, Frankfurt am Main, Germany; 4 Center of Experimental Orthopaedics and Department of Orthopaedic Surgery, Saarland University Medical Center, Homburg/Saar, Germany

**Keywords:** Intervertebral disc degeneration, Adipose-derived mesenchymal stem cells, Nucleus pulposus cells, Co-culture, Gene microarray analysis, Long non-coding RNAs, Micro RNAs

## Abstract

**Background:**

Stem cell therapy is considered as a promising alternative to treat intervertebral disc degeneration (IDD). Extensive work had been done on identifying and comparing different types of candidate stem cells, both in vivo and in vitro. However, few studies have shed light on degenerative nucleus pulposus cells (NPCs), especially their biological behavior under the influence of exogenous stem cells, specifically the gene expression and regulation pattern. In the present study, we aimed to determine messenger RNAs (mRNAs) and long non-coding RNAs (lncRNAs), which are differentially expressed during the co-culturing process with adipose-derived mesenchymal stem cells (ASCs) and to explore the involved signaling pathways and the regulatory networks.

**Methods:**

We compared degenerative NPCs co-cultured with ASCs with those cultured solely using lncRNA-mRNA microarray analysis. Based on these data, we investigated the significantly regulated signaling pathways based on the Kyoto Encyclopedia of Genes and Genomes (KEGG) pathway database. Moreover, 23 micro RNAs (miRNAs), which were demonstrated to be involved in IDD were chosen; we investigated their theoretic regulatory importance associated with our microarray data.

**Results:**

We found 632 lncRNAs and 1682 mRNAs were differentially expressed out of a total of 40,716 probes. We then confirmed the microarray data by real-time PCR. Furthermore, we demonstrated 197 upregulated, and 373 downregulated Gene Ontology terms and 176 significantly enriched pathways, such as the mitogen-activated protein kinase (*MAPK*) pathway. Also, a signal-net was constructed to reveal the interplay among differentially expressed genes. Meanwhile, a mRNA-lncRNA co-expression network was constructed for the significantly changed mRNAs and lncRNAs. Also, the competing endogenous RNA (ceRNA) network was built.

**Conclusion:**

Our results present the first comprehensive identification of differentially expressed lncRNAs and mRNAs of degenerative NPCs, altered by co-culturing with ASCs, and outline the gene expression regulation pattern. These may provide valuable information for better understanding of stem cell therapy and potential candidate biomarkers for IDD treatment.

**Electronic supplementary material:**

The online version of this article (10.1186/s13075-018-1677-x) contains supplementary material, which is available to authorized users.

## Background

Intervertebral disc degeneration (IDD) due to aging and other reasons is still a major clinical challenge for spine surgeons. Numerous strategies have been studied to prevent the deterioration and progressing of IDD, which will cause various pathologies, such as disc herniation, spinal stenosis and segmental instability [1]. However, disc degeneration is difficult to reverse in the natural course due to the avascular and aneural nature of the nucleus pulposus (NP). Biochemically, the degeneration is characterized by the decrease of the extracellular matrix and loss of water content within the NP, which are believed to play a crucial role in the progress of IDD.

Recently, stem cell therapy, introducing mesenchymal stem cells (MSCs) or cytokines into target discs, appears as a promising strategy for the IDD treatment. This emerging modality was proved to be effective both in vitro [2–4] and in vivo [5, 6]. Co-culturing with nucleus pulposus cells (NPCs), MSCs could differentiate towards the NP-like cells with increased expression of the NP marker genes [7]. In particular, adipose-derived mesenchymal stem cells (ASCs) co-cultured with NPCs expressed significantly upregulated expression of NP-related genes including sex determining region Y box 9 (*SOX9*), type II collagen (*COL2A1*), and aggrecan (*ACAN*) [8]. Interestingly, our previous study demonstrated that degenerative NPCs could be activated by MSCs in the co-culture system with significantly upregulated gene expression of *SOX9, COL2A1*, and *ACAN* [4]. It is notable that degenerative NPCs could be induced towards healthy NPCs by specific cytokines and cellular factors yielded from MSCs [9]. Also, it underlines that the gene expression pattern of not only MSCs but also degenerative NPCs could be altered through the intracellular cross-talking [10, 11]. However, the underlying mechanism of these phenomena is still not fully understood.

Gene microarray technology can simultaneously measure differences in the expression level of thousands of genes of predefined groups of samples [12] and allows highly effective evaluation of genome-wide expression changes [13]. This novel technology enables researchers to develop a more comprehensive understanding of the cross-talking between MSCs and degenerative NPCs, including differentially expressed genes and long non-coding RNAs (lncRNAs).

Recently, lncRNAs have received critical attention with their regulatory effect on gene expression [14, 15]. They have been characterized with the high tissue-specificity and low sequence-conservation [16, 17] and have been demonstrated to be involved in various physiological and pathological processes as regulators, such as imprinting, X-inactivation, and development [18]. Numerous previous studies have attempted to map the phenotype of NPCs [19–22], to the best of our knowledge, yet few studies have emphasized the function of lncRNAs related to disc degeneration, particularly their regulatory roles in the process of degenerative NPCs co-cultured with MSCs [23].

Similarly, micro RNAs (miRNAs), a group of small and non-coding RNAs, have been proved to participate in the expression regulation of coding genes and to influence various biological processes, including cell differentiation, proliferation, and metabolism [15, 24]. Many miRNAs have been demonstrated to be involved in natural disc degeneration [25, 26], and specifically *miR-27b* [27] and *miR-93* [28] have been shown to promote matrix degradation within the discs and accelerate the disc degeneration process. Yet, there was no study focusing on the role of these miRNAs in the cell-cell cross-talk between MSCs and degenerative NPCs.

The current study aimed to use gene expression microarray analysis and bioinformatics methods to investigate the effect of MSCs on degenerative NPCs in terms of deferentially expressed lncRNAs and mRNAs, signaling pathways, and gene regulation networks involving mRNAs, lncRNAs, and miRNAs (Additional file 1).

## Methods

All human tissues were obtained and used with informed consent from the patients and under the approval of the Institutional Review Broad of the Shanghai General Hospital, Shanghai Jiaotong University.

### Isolation and culture of NP cells

The human NP tissue was surgically obtained from the degenerative discs (grade III–IV according to the Pfirrmann grading system [29]) of three patients diagnosed as having lumbar spondylosis. The isolation and culture of NPCs was performed as previously reported [4, 30] (Additional file 2).

### Isolation and culture of ASCs

The adipose tissue surgically obtained from the patients’ backs was processed for isolation of ASCs following the previously standardized protocol [31, 32] (Additional file 2).

### Co-culture of ASCs and NPCs

Both NPCs and ASCs at passage 3 were co-cultured using the non-direct cell-cell contact co-culturing system, consisting of six-well plates and polyethylene terephthalate track-etched tissue culture inserts with 0.4-μm pore size. Briefly, ASCs (6.0 × 10^4^ cells) were seeded on the base of the six-well plate, and the same numbers of NPCs were seeded onto the upper surface of the membrane. Co-cultured cells were maintained for 7 days at 37 °C and 5% CO_2_ in a humidified atmosphere with the medium being changed every 2 days. Meanwhile, the NPCs (6.0 × 10^4^ cells) at passage 3 were cultured for 7 days in the same condition as the control.

### RNA isolation and quality control

Briefly, the total RNA was isolated from each group of cells using the Trizol agent (Invitrogen, Carlsbad, USA) following the manufacture’s protocol. Then the RNA was purified with an RNase Kit (Bio-Rad, CA, USA), and the quantity was measured using a spectrophotometer (NanoDrop-1000, Thermo Scientific, MA, USA). Agarose-gel electrophoresis was performed to test the RNA integrity and DNA contamination (Additional file 3).

### Microarray analysis

Generally, NPCs without co-culture (control group) and with co-culture (experimental group) were used to compare mRNA and lncRNA expression profiles. As shown in additional file 1, a multiple-step strategy was used to identify mRNAs and lncRNAs dysregulated between NPCs with and without co-culturing. To clarify the changes in the signaling pathways of NPCs during co-culture, we further performed Gene Ontology (GO) analysis, pathway analysis, and signal-net analysis. Microarray analysis was performed by the GMINIX Informatics Ltd. Co (Shanghai, China). The quality control of hybridization is shown in Additional file 4. The data had been uploaded to the NCBI Gene Expression Omnibus (GEO) and can be accessed [GEO:GSE112216] (https://www.ncbi.nlm.nih.gov/geo/query/acc.cgi?acc=GSE112216).

The mRNAs and lncRNAs differently expressed between NPCs with and without co-culturing were identified using the random variance model (RVM) *t* test. The RVM *t* test was applied to filter the differentially expressed RNAs with increased degrees of freedom in the small sample datasets. With a threshold of *P* < 0.05 considered significant, differentially expressed RNAs were selected and false discovery rate (FDR) analysis was performed. Unsupervised hierarchical clustering was performed and a cluster map was created.

### Real-time PCR

To validate the microarray results, seven mRNAs were selected for the real-time PCR validation. Complementary DNA (cDNA) was generated by the reverse transcript using a Taqman Reverse Transcription Kit (Invitrogen, Carlsbad, USA) according to the manufacturer’s instructions. Gene expression analysis was conducted by real-time PCR using the SYBR Green Mastermix (BioRad, CA, USA) and a CFX96 Touch Real-time PCR Detection System (BioRad, CA, USA). Homo actin was used as the internal control to determine the relative expression of target genes; the relative changes in gene expression were compared to those of untreated cells using the 2^-ΔΔCT^ method where CT = threshold cycle. All reactions were performed in triplicate and the sequences of used primers are shown in Table 1.

**Table 1:** Primers used in real-time PCR

Gene name	Forward sequence 5′-3′	Reverse sequence 5′-3’
*PIK3R3*	GGG GAA GTG AAG CAC TGT GT	GAC GTT GAG GGA GTC GTT GT
*ENPP1*	GCC CGA AAT CTT TCT TGC CG	TGC CAT GCT TGA ATC CAG GT
*MT1F*	TGC AAG TGC AAA GAG TGC AA	CCC TTT GCA AAC ACA GCC C
*SPP1*	GCC GAG GTG ATA GTG TGG TT	AAC GGG GAT GGC CTT GTA TG
*EPYC*	TTC TGG GGC CAC ACA CAA AT	GCT CTC GAA GTT GAG GCA GT
*CD24*	GCT CCT ACC CAC GCA GAT TT	GAG ACC ACG AAG AGA CTG GC
*C4orf31*	TCA TGT CTA CTC CAG GCC CA	GTA GTA CTG CGT GTC GGG TT
*homo-actin*	GCT CAG GAG GAG CAA TGA TCT TG	GTA CGC CAA CAC AGT GCT GTC

*PIK3R3* phosphoinositide-3-kinase, regulatory subunit 3, *ENPP1* ectonucleotide pyrophosphatase/ phosphodiesterase 1, *MT1F* metallothionein 1F, *SPP1* secreted phosphoprotein 1, *EPYC* epiphycan, *C4orf31* chromosome 4 open reading frame 31

### GO analysis

Based on the GO database (http://www.geneontology.org), the GO analysis was performed to analyze the main functions of the differentially expressed mRNAs using the two-sided Fisher’s exact test and chi-square test. The differentially expressed genes were evaluated independently and classified to upregulation and downregulation. *P* values of all differentially expressed genes were computed in all GO categories, and *P* < 0.01 was defined as significant.

### Pathway analysis

The significance levels of pathways associated with differentially expressed genes were analyzed based on the Kyoto Encyclopedia of Genes and Genomes (KEGG) database (http://www.genome.jp/kegg/). Fisher’s exact test and the chi-square test were used to select the significant pathways according to the significance threshold *P* < 0.05.

In order to systematically identify the integrations between the pathways, the Path-net, highlighting the interaction net containing the pathways associated with differentially expressed genes, was generated based on the interactions among the pathways of the KEGG database.

### Signal-net analysis

The significant intersectional genes in both GO analysis and Pathway analysis were selected to analyze the gene-gene interaction and construct the network map. From the differentially expressed gene data, the gene-gene network map was constructed based on the KEGG database, allowing the users to build and analyze the molecular networks. The networks are stored and presented as graphs, the nodes are genes, and edges representing relationship types between the nodes may indicate activation or phosphorylation. The graph nature of networks allowed further investigation with the powerful tools implemented in R. The network work of each gene was calculated by counting the numbers of upstream genes and downstream genes, which were expressed in the form of in-degree and out-degree. The betweenness centrality of each gene was calculated according to its in-degree and out-degree, and higher betweenness centrality implies greater importance in the gene-network regulation.

### LncRNA-gene-net analysis

Co-expression network analysis of lncRNAs and mRNAs was performed based on the differentially expressed lncRNAs and the intersectional mRNAs, which were significant in both GO analysis and Pathway analysis.

### Competing endogenous RNA (ceRNA) analysis

The miRNAs are a class of ~ 22-nucleotide-long single-stranded non-coding RNAs that regulate gene expression by binding to miRNA response elements (MREs) on the RNAs [33, 34]. The lncRNAs are non-coding RNAs longer than 200 nucleotides and also involved in the pathology of many complex human diseases including cancer [35]. The lncRNAs also harbor MREs and compete with other RNAs for the miRNA binding, and lncRNAs can regulate miRNA abundance by sequestering and binding them [36], thus functioning to compete with endogenous RNAs to influence post-transcriptional regulation.

Based on previous studies, 23 miRNAs associated with disc degeneration (Additional file 5) were selected to investigate their regulatory involvement in differentially expressed mRNAs and lncRNAs defined by our study. Then the miRNA-mRNA target prediction according to TargetScan (http://www.targetscan.org/) and the miRanda (http://cbio.mskcc.org/miRNA2003/miranda.html) was performed for competing endogenous RNA. The ceRNA network was thereafter constructed based on those negatively regulated intersectional lncRNAs and mRNAs.

### Statistical analysis

All data are reported as mean ± standard deviation. Differences between groups were evaluated by Student’s *t* test using SPSS 20.0 software (Chicago IL, USA.). *P* < 0.05 was considered statistically significant.

## Results

### Validation of ASCs

**Fig. 1:**
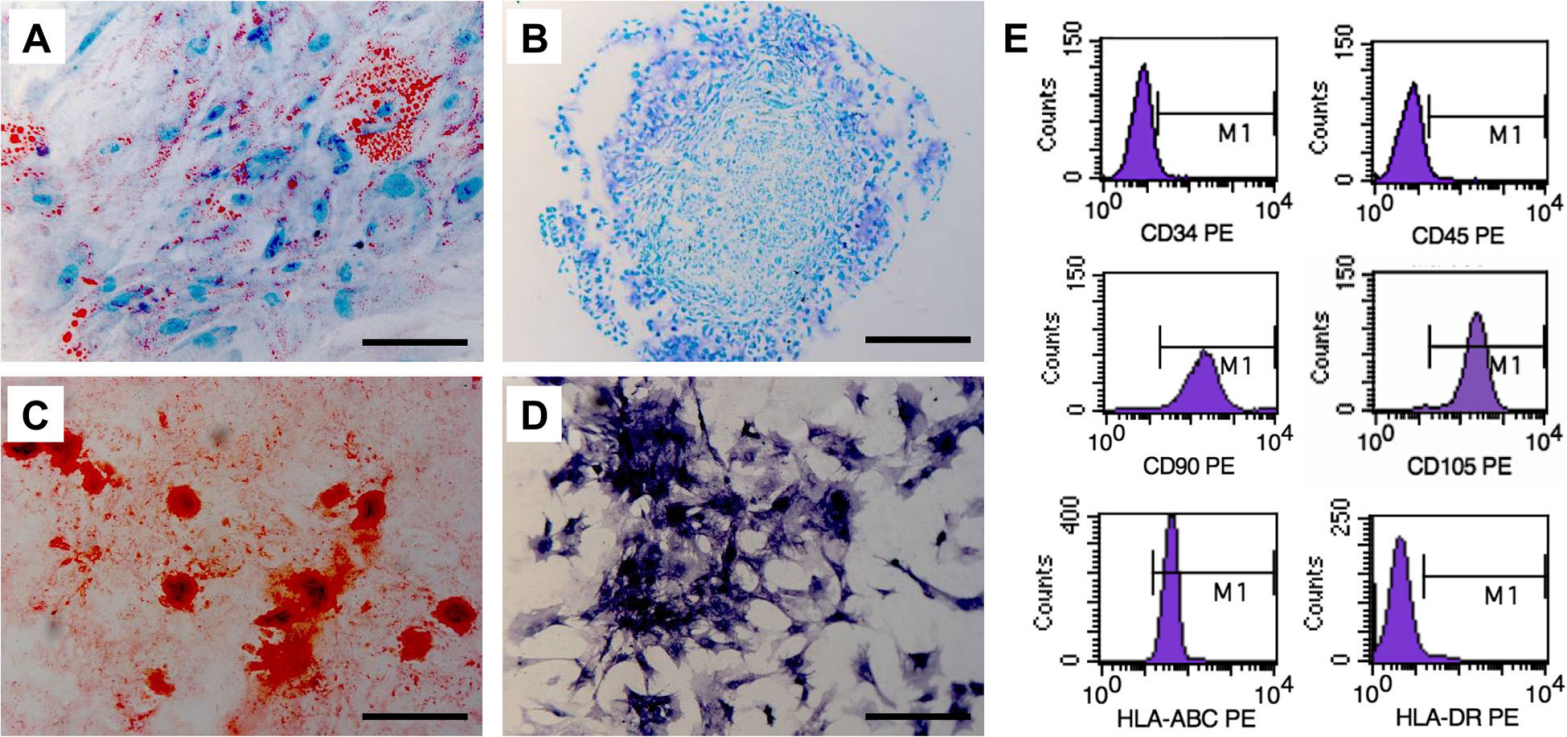
Identification of adipose-derived mesenchymal stem cells (ASCs). **a-d** Multilineage differentiation ability of the ASCs, verified by histological assays. **a** Oil Red O staining showed the adipogenic differentiation potential of the ASCs. The red dots (indicated by arrows) stand for the differentiated adipocytes colored by Oil Red O staining. **b** Alcian Blue staining for chondrogenic differentiation. The blue deposits were indicative of functional chondrocytes. The osteogenic differentiation verified by Alizarin Red (**c**), the calcium deposits were stained red (indicated by arrows) and the osteoblasts were in dark blue stained by alkaline phosphatase staining (**d**). **e** Expression of specific surface markers of ASCs by flow cytometry. The ASCs positively expressed CD90, CD105 and HLA-ABC and negatively expressed CD34, CD45 and HLA-DR

To verify the ASCs, specific surface markers and multiple differentiation potentials were verified. On flow cytometry, positive expression of CD90, CD105, and HLA-ABC and negative expression of CD34, CD45, and HLA-DR was observed (Fig. 1). Also, the multilineage differentiation potential of ASCs was proved by the histological staining (Fig. 1).

### Identification of deferentially expressed lncRNAs and mRNAs

**Fig. 2:**
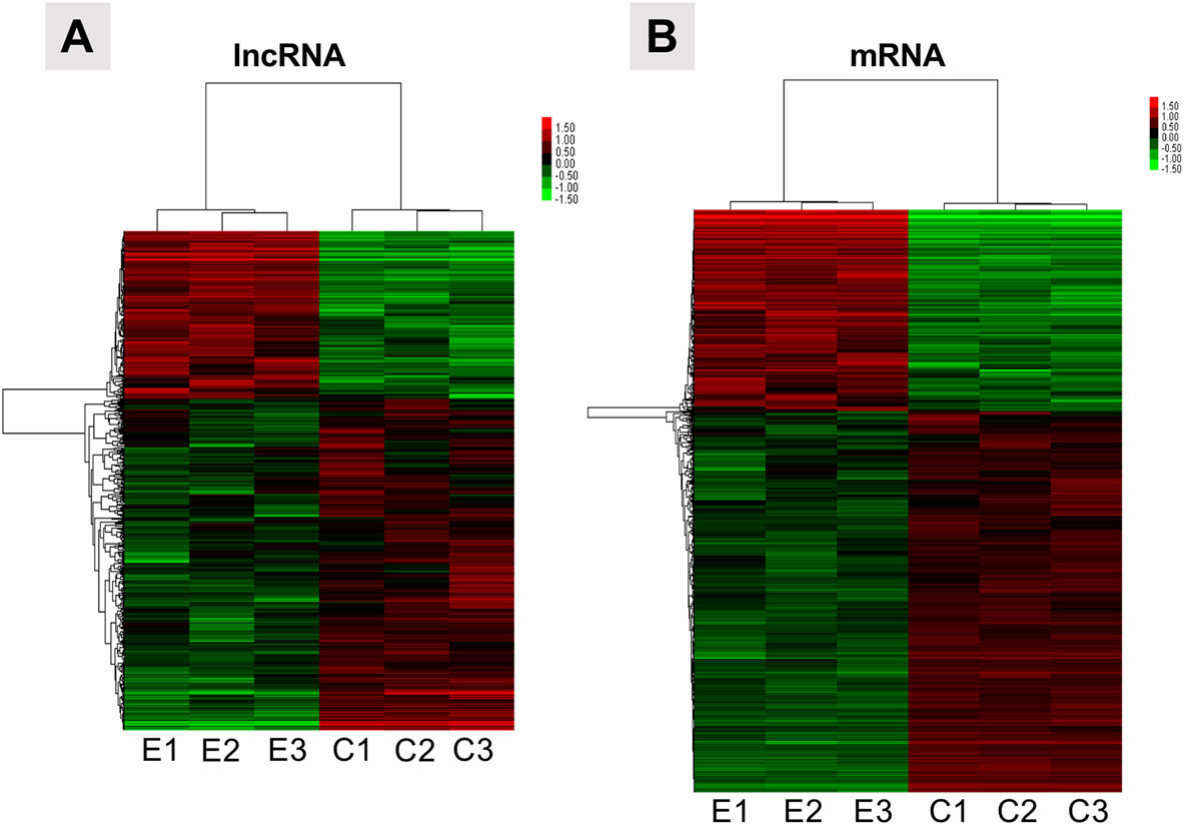
Cluster heat map shows differentially expressed long noncoding RNAs (lncRNAs) and messenger RNAs (mRNAs). The names of the sample groups are on the x-axis and the different probles are on the y-axis. The red strip indicates high relative expression and the green strip indicates low relative expression. E, experimental group with nucleus pulposus cells (NPCs) from degenerative discs, which were co-cultured with adipose-derived mesenchymal stem cells (ASCs); C, control group with NPCs from degenerative discs not co-cultured with ASCs

A total of 2314 probes were identified to be differentially expressed, including 632 lncRNAs and 1682 mRNAs (Fig. 2a). For lncRNAs, the chromosome-3 open reading frame 49 (*C3orf49*) showed the greatest upregulation (fold change = 18.9), followed by the two-pore channel 3 pseudogene, upregulated (*LOC440895*) and testis-specific transcript Y-linked 15, upregulated (*TTTY15*). Additionally, the top three deferentially expressed mRNAs were the secreted phosphoprotein 1 (*SPP1*), metallothionein 1F (*MT1F*) and ectonucleotide pyrophosphatase 1 (*ENPP1*) with their fold changes were 106, 77, and 34, respectively (Fig. 2b). Furthermore, these deferentially expressed mRNAs also include NPCs maker mRNAs such as *SOX-9 *and *COL2A1*. More detailed information is provided in Additional file 6 (top 10 differentially expressed lncRNAs and mRNAs) and Additional file 7 (full list of differentially expressed mRNAs and lncRNAs).

### Validation of real-time PCR

**Fig. 3:**
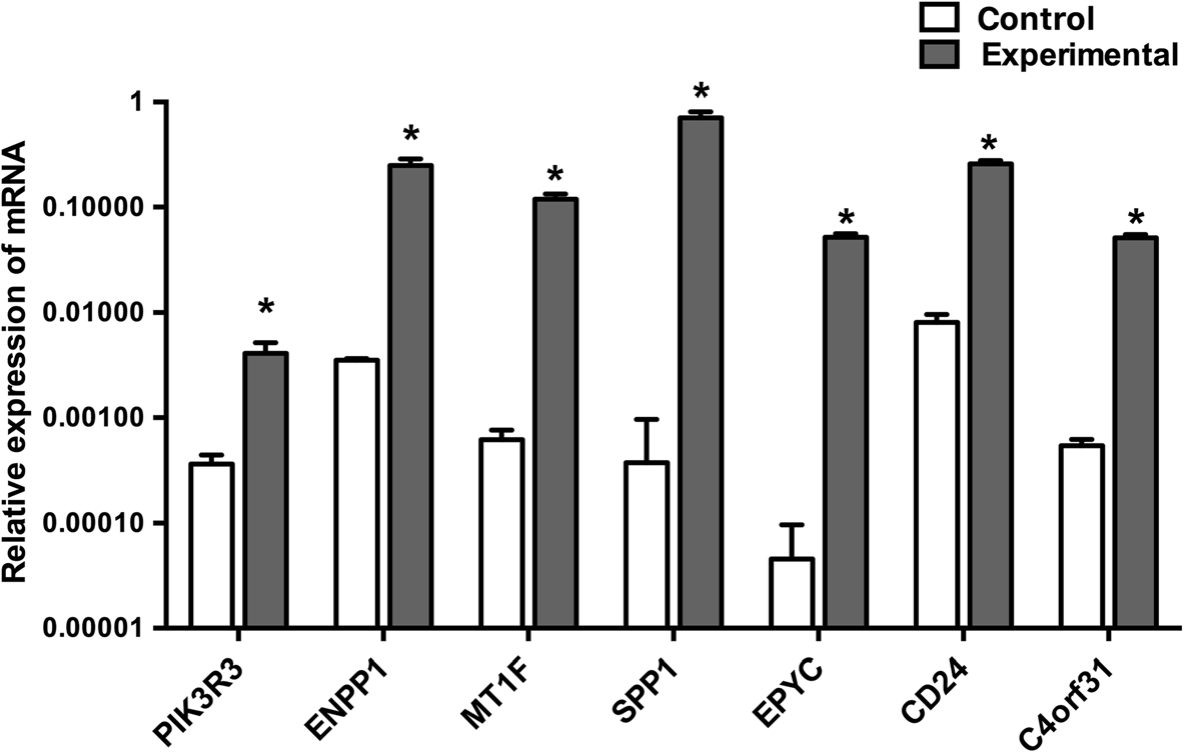
Real-time PCR confirmation of gene microarray results. Seven genes (*PIK3R3*, *ENPP1*, *MT1F*, *SPP1*, *EPYC*, *CD24*, *C4orf31*) were selected for the real-time PCR validation. Control represents nucleus pulposus cells (NPCs) from degenerative discs not co-cultured with adipose-derived mesenchymal stem cells (ASCs); experimental represents NPCs after co-culturing with ASCs. *P < 0.05 compared with control group

Furthermore, we demonstrated that the results of the real-time PCR analysis were consistent with the microarray data. Seven differentially expressed mRNAs (*PIK3R3, ENPP1, MT1F, SPP1, EPYC, CD24, *and *C4orf31*) were selected for the real-time PCR analysis. The gene chip analysis revealed these mRNAs were upregulated up to 2.70-fold, 34.48-fold, 76.92-fold, 106.38-fold, 34.58-fold, 20.40-fold, and 17.85-fold, respectively. The microarray analysis data were also verified, and the expression of *PIK3R3 *(*P* < 0.05), *ENPP1* (*P* < 0.05), *MT1F* (*P* < 0.05), *SPP1* (*P* < 0.05), *EPYC* (*P* < 0.05), *CD24 *(*P* < 0.05), *C4orf31 *(*P* < 0.05) in the NPCs were significantly increased up to 11.32-fold, 70.92-fold, 192.01-fold, 1896.14-fold, 1132.71-fold, 31.87-fold, and 94.45-fold, respectively (Fig. 3).

### GO analysis

**Fig. 4:**
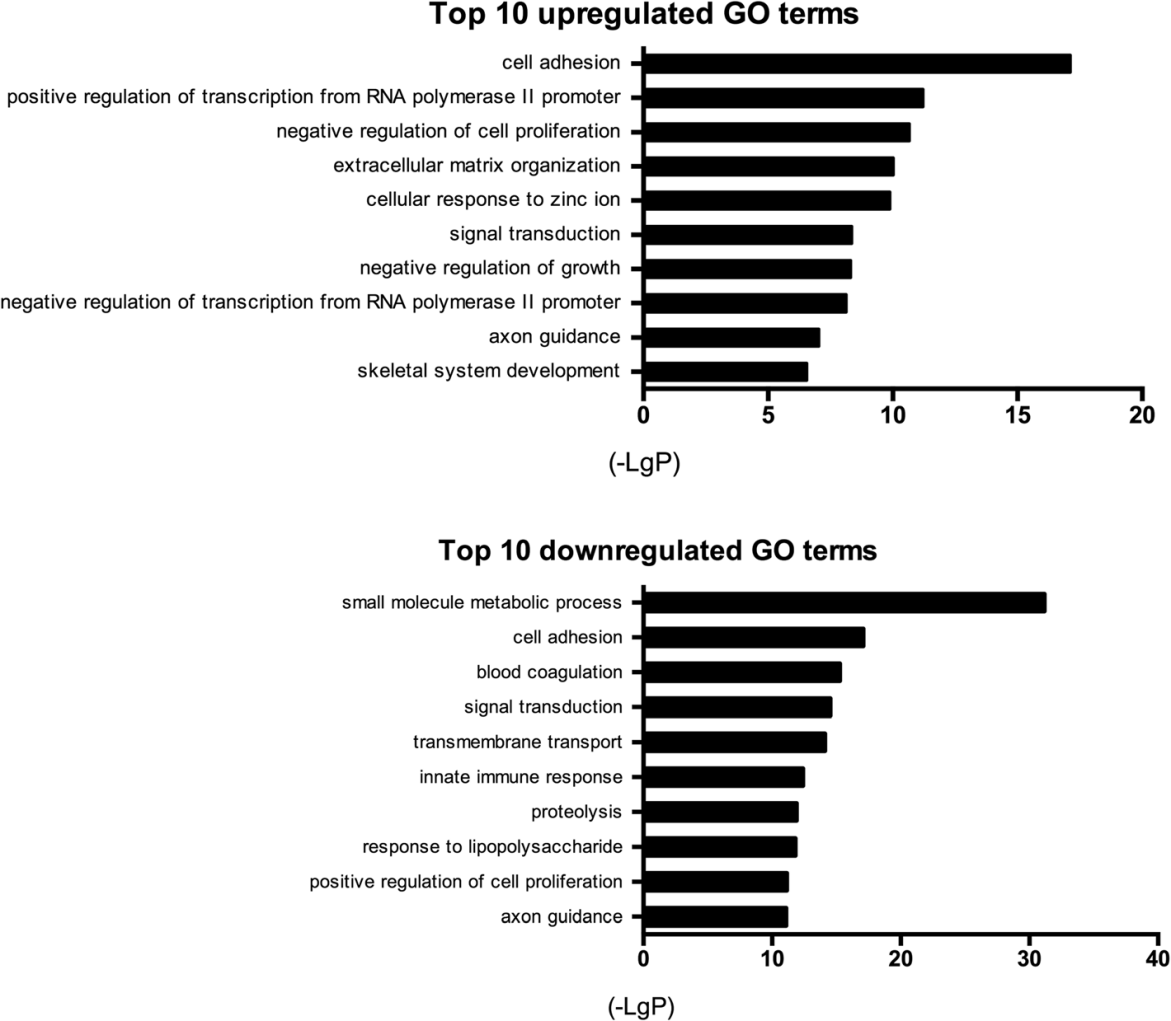
Top 10 significantly upregulated and downregulated Gene Ontology (GO) terms. The length of the bars on the x-axis represents the negative logarithm of the *P* value (-LgP) of each GO term; higher -LgP values indicate higher significance and lower -LgP value indicate lower significance. The GO term names are shown on the y-axis

Differentially expressed mRNAs and lncRNAs were used for the downstream GO analysis and pathway analysis. There were 197 upregulated and 373 downregulated GO terms (*P* < 0.05), including the upregulated cell adhesion (GO:0007155) and the downregulated small molecule metabolic process (GO:0044281). The top 10 upregulated and downregulated GO terms are presented in Fig. 4.

### Pathway analysis

**Fig. 5:**
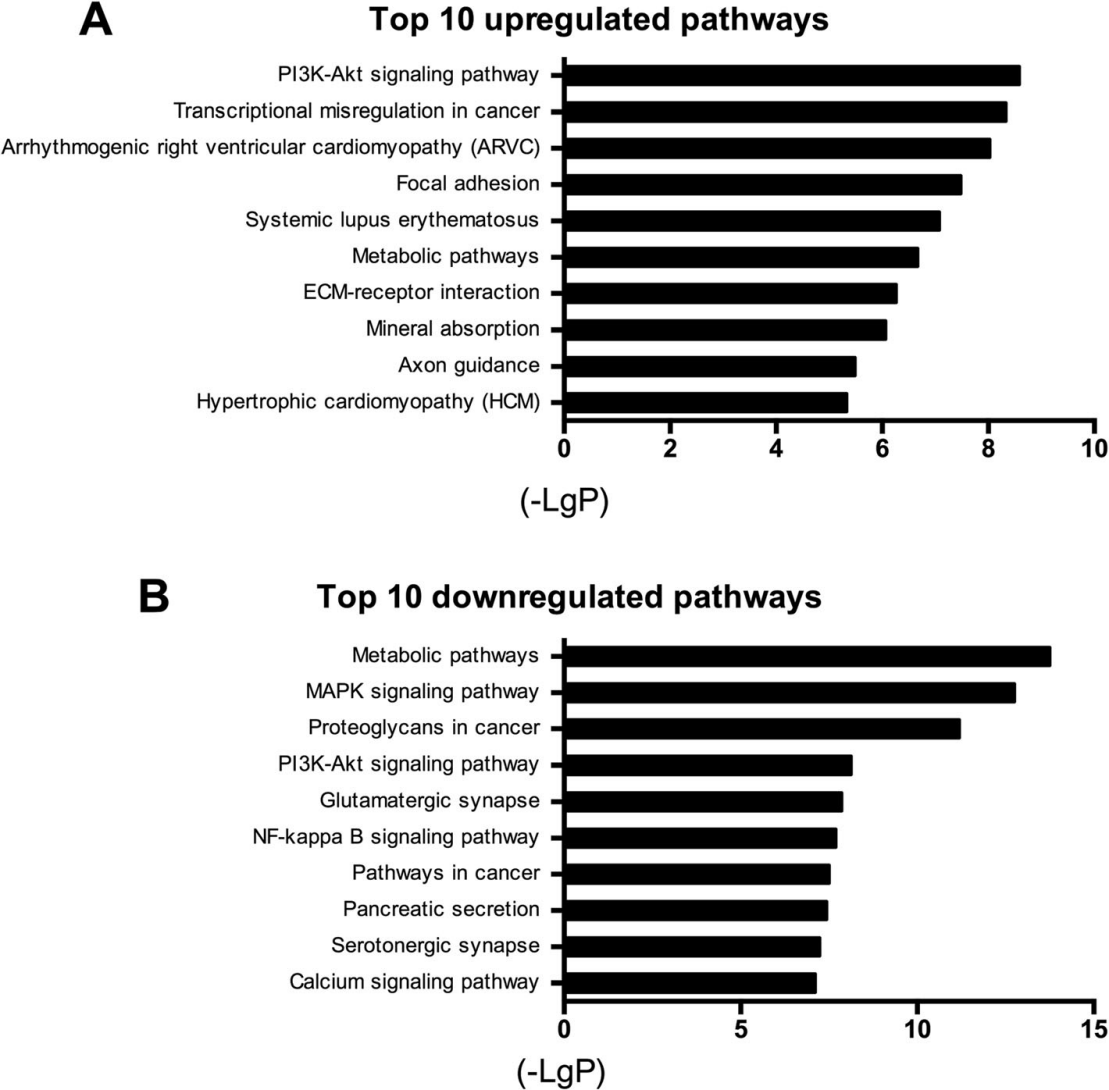
Results of the Kyoto Encylopedia of Genes and Genomes (KEGG) pathway analysis. **a** Top 10 most significantly upregulated KEGG pathways. **b** Top 10 significantly downregulated KEGG pathways. The length of the bars on the x-axis represents the negative logarithm of the *P* value (-LgP) of each pathway; higher -LgP values indicate higher significance and lower -LgP values indicate lower significance. The pathway names are shown on the y-axis. ECM, extracellular matrix; MAPK, mitogen-activated protein kinase

The pathway analysis identified a total of 176 pathways, among which 122 pathways were statistically significantly upregulated and 54 were significantly downregulated. The most enriched pathways included phosphoinositide 3-kinase (PI3K)-protein kinase PI3K-Akt signaling pathway (upregulated) and metabolic pathways (downregulated) (Fig. 5).

**Fig. 6:**
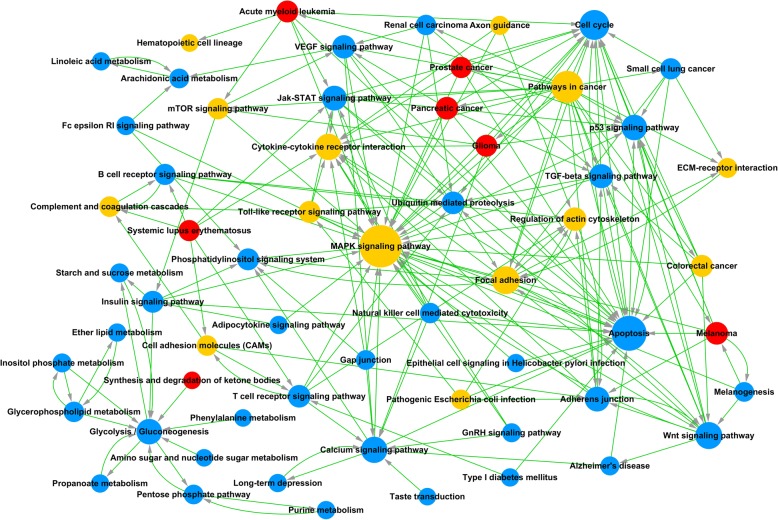
Path-net analysis of differentially signaling pathways. Nodes represent different pathways; red indicates upregulated, blue indicates downregulated, and yellow indicates both upregulated and downregulated. The size of each circle is determined by the number of other genes that interact with this gene, namely the degree of the pathway. ECM, extracellular matrix; MAPK, mitogen-activated protein kinase; VEGF vascular endothelial growth factor; TGF, transforming growth factor

The interaction network for all significantly enriched pathways is formulated with the Path-net (Fig. 6; Additional file 8), representing these pathways directly and systematically involved in the interaction between ASCs and NPCs. Also, we discovered that the mitogen-activated protein kinase (MAPK) signaling pathway played a canonical role with the highest number of interactions with other pathways (interaction degree, 35) (Fig. 6). In contrast, the apoptosis pathway (interaction degree, 25) and the Wnt signaling pathway (interaction degree, 18) were downregulated in the co-culture system.

### Signal-net

**Fig. 7:**
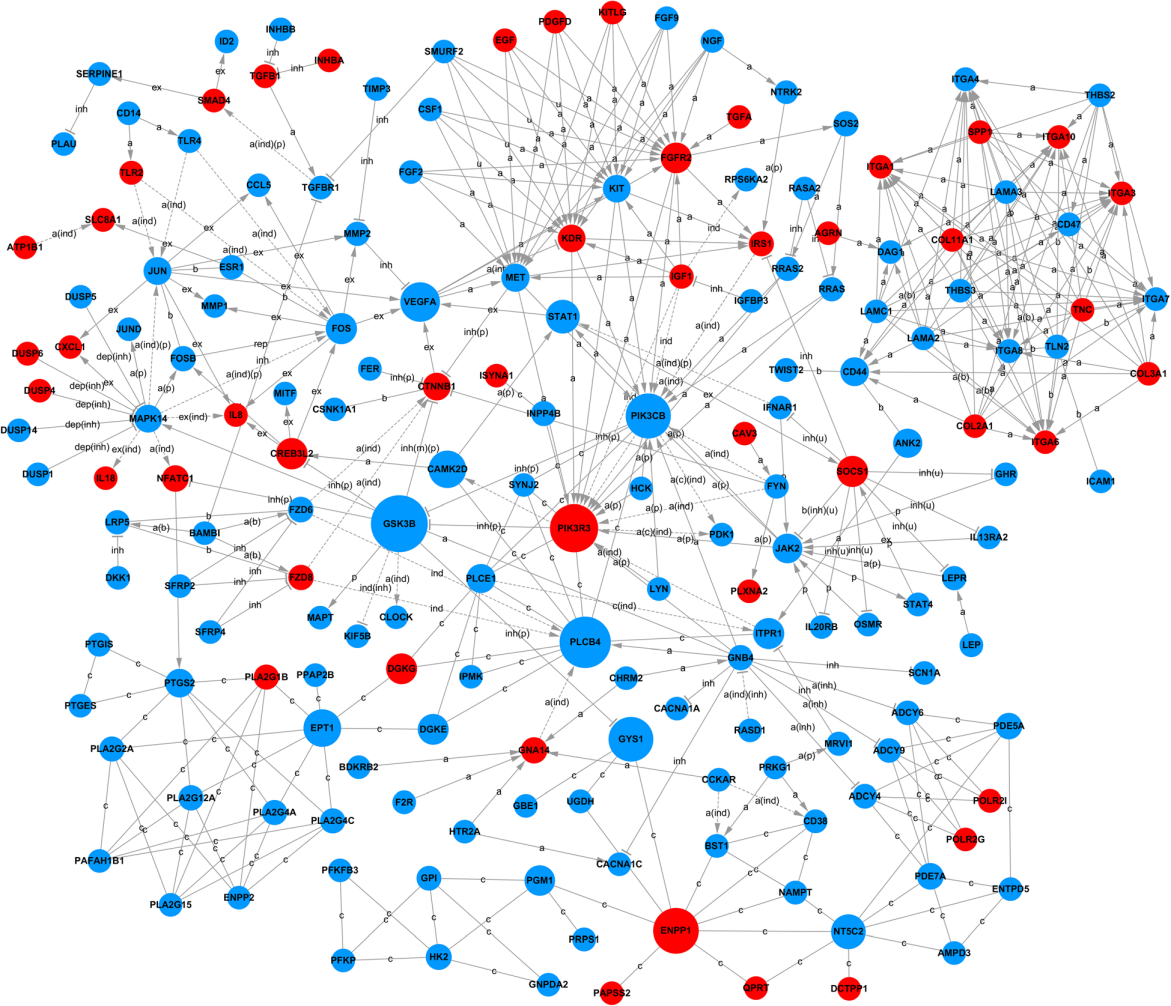
Signal-net analysis of the differentially expressed genes between the control and experimental group. Circles represent genes, red indicates upregulated and blue indicates downregulated. The size of each circle represents the number of the other genes that interact with this gene. Lines indicate interactions between genes. The relation between two genes is indicated as a(b), activation (binding/association); a(c)(ind), activation (compound) (indirect effect); a(ind), activation (indirect effect); a(ind)(inh), activation (indirect effect) (inhibition); a(ind)(p), activation (indirect effect) (phosphorylation); a(inh), activation(inhibition); a(p), activation (phosphorylation); b, binding / association; b(inh)(u), binding / association (inhibition) (ubiquitination); c, compound; c(ind), compound (indirect effect); dep(inh), dephosphorylation (inhibition); ex, expression; ex(ind), expression (indirect effect); ind, indirect effect; ind(inh), indirect effect (inhibition); inh, nhibition; inh(m)(p), inhibition (missing interaction) (phosphorylation); inh(p), inhibition(phosphorylation); inh(u), inhibition (ubiquitination); p, phosphorylation; rep, repression; u, ubiquitination

Based on the intersectional genes that were significantly enriched in both GO analysis and Pathway enrichment analysis, a gene interaction network was constructed (Fig. 7; Additional file 9). Signal-net analysis showed that phosphoinositide-3-kinase, regulatory subunit 3, upregulated (*PIK3R3*), phosphoinositide-3-kinase, catalytic, beta polypeptide, downregulated (*PIK3CB*) and fibroblast growth factor receptor 2, downregulated (*FGFR2*) are critical in the gene regulatory network with their degrees of connections 19, 18 and 15, respectively.

### lncRNA-mRNA co-expression network

**Fig. 8:**
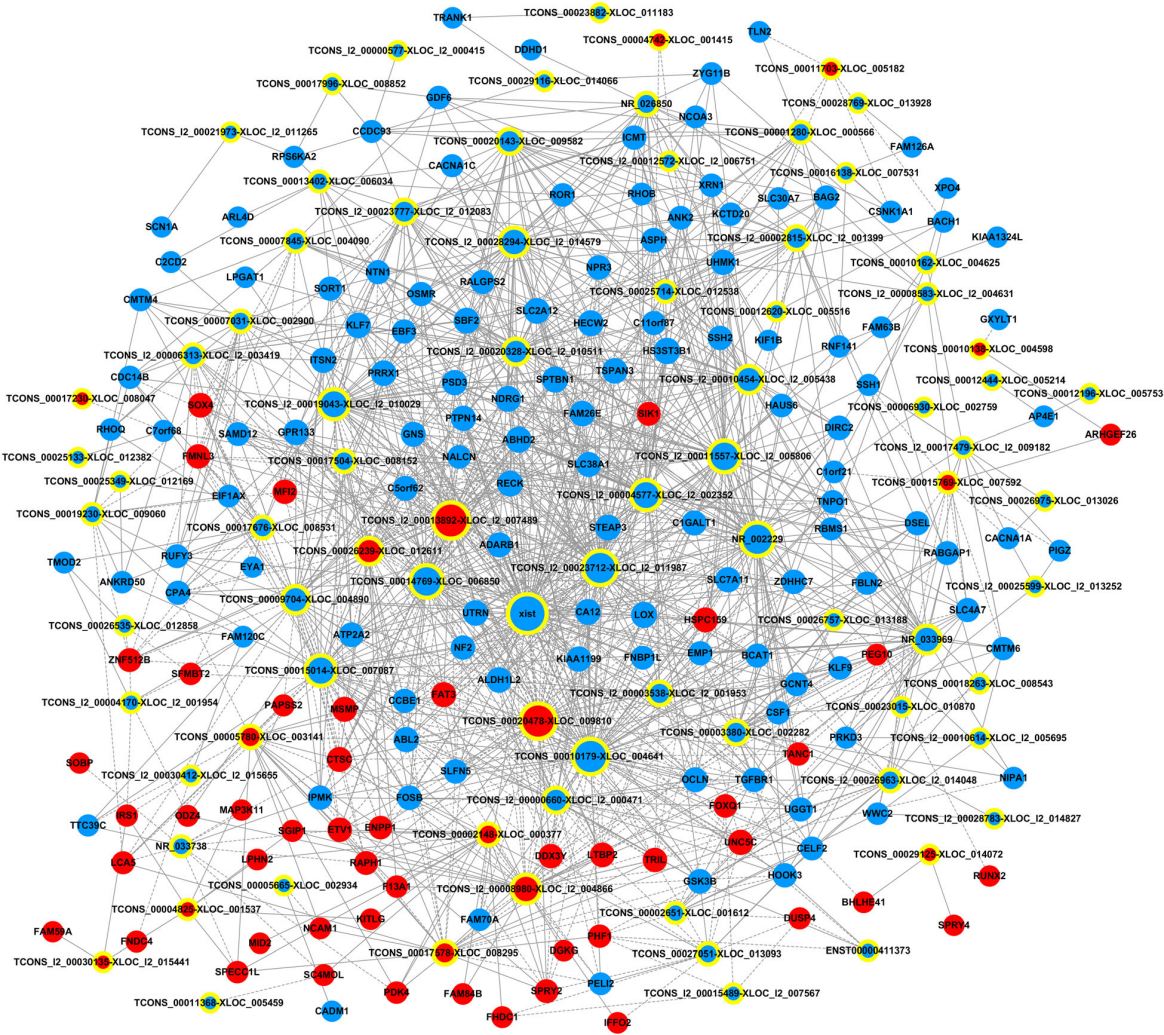
The long noncoding RNA (lncRNA)-messenger RNA (mRNA) co-expression network of the 80 lncRNAs and 170 mRNAs. A node without a yellow ring indicates mRNA, and a node with a yellow ring indicates lncRNA. Upregulated mRNAs and lncRNAs are shown in red and downregulated mRNAs and lncRNAs are shown in blue. A solid line indicates positive interaction; a dashed line indicates negative interaction

A co-expression network was built for 80 lncRNAs and 170 mRNAs selected from the differentially expressed lncRNAs and mRNAs based on the degree of correlation (Fig. 8). These 250 RNAs (250 nodes in the co-expression network) were further combined into 1453 pairs of co-expression lncRNA-mRNA. Among these RNAs of the co-expression network, X-inactive specific transcript, (non-coding, downregulated, degree, 77 (*XIST*) obtained the highest number of interactions followed by the TCONS_l2_00013892-XLOC_l2_007489 (non-coding, upregulated, degree, 69) and TCONS_00020478-XLOC_009810 (non-coding, upregulated, degree, 66).

### Regulatory role of miRNAs

**Fig. 9:**
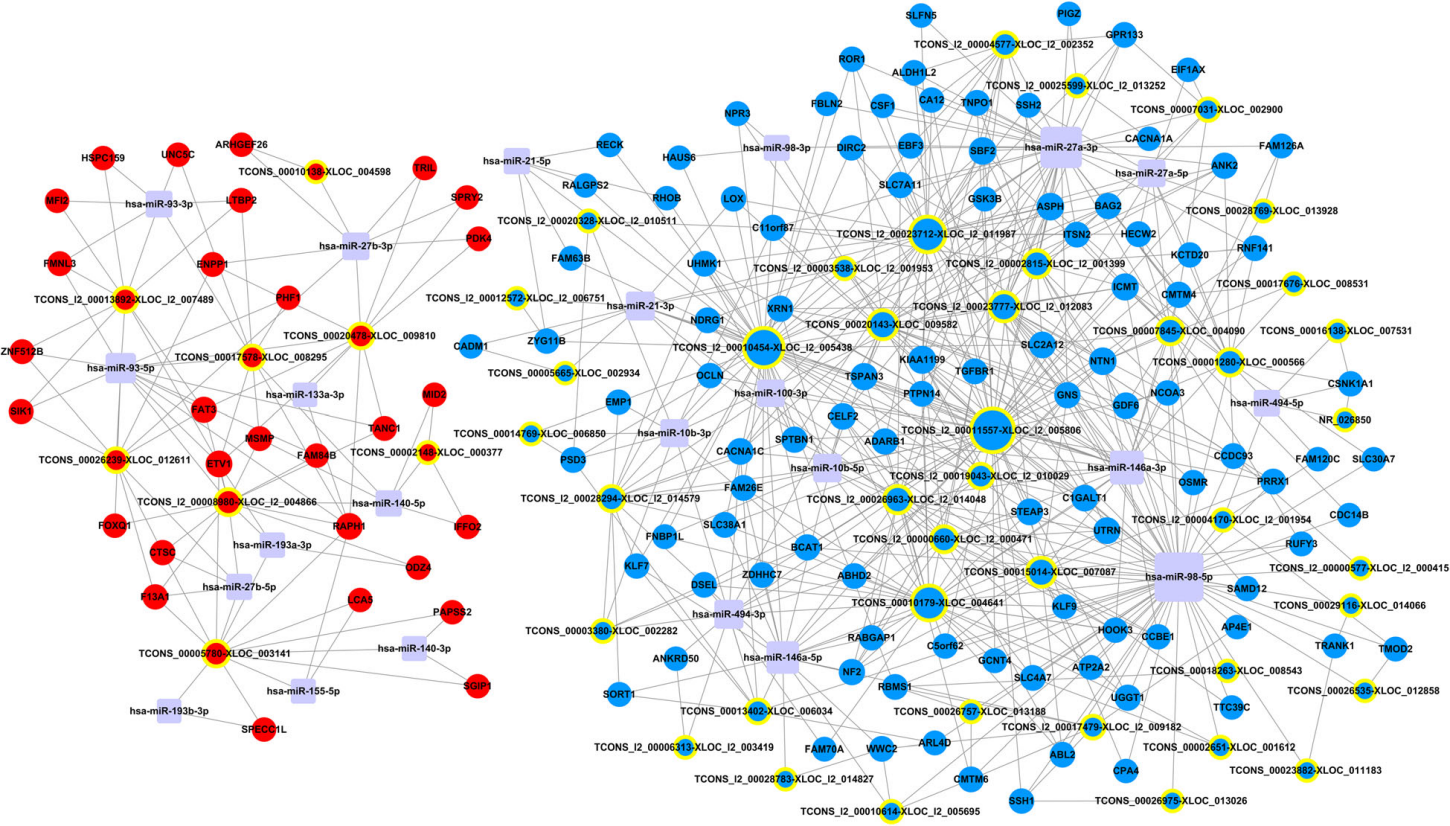
The long noncoding RNA (lncRNA)-messenger RNA (mRNA)-micro RNA (miRNA) competing endogenous RNA (ceRNA) network. Nodes with a yellow ring represent lncRNAs; nodes without a yellow ring represent mRNAs; rectangles with rounded corners represent miRNAs. Upregulated RNAs are shown in red and downregulated RNAs are shown in blue

A competing endogenous RNA network was constructed from 23 miRNAs, previously proved to be involved in intervertebral disc degeneration, and displayed their regulatory interplay with differentially expressed mRNAs and lncRNAs (Fig. 9, Additional file 5). Evidently, hsa-miR-98-5p (upregulated, interaction degree, 57) was the most important miRNA in this network, followed by the hsa-miR-27a-3p (upregulated, interaction degree, 40) and hsa-miR-146a-3p (upregulated, interaction degree, 23). Additionally, TCONS_l2_00011557-XLOC-_l2_005806 TCONS_l2_00010454-XLOC_l2_005438, and TCONS_l2_00023712-XLOC_l2_011987 were the top three important lncRNAs in this regulatory network; their interaction degrees were 53, 44, and 35, respectively (Additional file 6 and Additional file 7).

## Discussion

In the present study, we identified a significant number of mRNAs and lncRNAs differentially expressed by degenerative NPCs co-cultured with ASCs, using the microarray analysis and in-depth data profiling. We also identified the signaling pathways that were altered during the co-culture and outlined the co-expression relationship between mRNAs and lncRNAs. The disc-degeneration-related miRNAs, differentially expressed mRNAs, and lncRNAs were further evaluated to identify the regulatory interaction, highlighting the biological pathways and cellular events of gene expression and regulation during the stem-cell therapy process. In addition, our research validates the previous studies about NPC phenotypes [19–22] and further investigated the regulatory role of lncRNAs. However, more in-depth understanding of these gene expression and regulation profiles will provide valuable clues for gene therapy approaches for disc degeneration.

In the present study, we revealed the altered mRNAs and lncRNAs between NPCs before and after co-culture with ASCs. Real-time PCR results of seven randomly selected mRNAs (Fig. 3) were consistent with the microarray data, further confirming the high credibility of the microarray analysis. Furthermore, we identified that the NP marker gene expression, *SOX-9* (fold change = 7, *P* < 0.001) and *COL2A1* (fold change = 6, *P* < 0.001), was increased in NPCs after the co-culture, indicating the upregulated synthesis and secretory activities of NPCs under the influence of MSCs [37, 38]. These findings were consistent with the previous studies reporting the significantly raised expression of *SOX-9* and *COL2A1* in NPCs when co-cultured with MSCs [39, 40].

In particular, we demonstrated that *SPP1* was the most significantly altered gene and might be indicated as a marker of NPC (Additional file 6). SPP1, also known as osteopontin (OPN), is an extracellular structural protein secreted by various types of cells. Our data from the signal-net analysis suggested that SPP1 could activate the integrin proteins, e.g. integrin alpha (ITGA) 1, 3, 4, 6, 7, 8, and 10, and has close connections with COL11A1, COL3A1, and COL2A1 (Fig. 7). Since integrins are a class of cell adhesion molecules that regulate interactions between a cell and its surrounding matrix [41], activation of ITGA will assist NPCs to interact with the extracellular matrix, specifically collagen molecules, and then to potentially restore the intervertebral disc function. Marfia and colleagues previously reported greater expression of *SPP1/OPN* and CD44 in degenerative IVD comparing to herniated IVD, and SPP1/OPN was only detected in degenerative IVD tissue [42]. They therefore assumed that SPP1/OPN might mark the severity of disc degeneration. However, in disagreement with Marfia’s study, our data revealed that SPP1/OPN was upregulated with downregulated CD44 when degenerative NPCs were co-cultured with ASCs (Additional file 7). Such a considerable divergence warrants further in-depth investigations into the sophisticated underlying mechanism.

We also demonstrated that CD24 were upregulated (Additional file 6) and these data were in accord with previous studies, signifying CD24 as an important marker of NPCs and notochordal cells [22, 43–48]. Particularly, Ricardo Rodrigues-Pinto et al. demonstrated that CD24, a glycosylphosphatidylinositol anchor protein, is one of the notochord-specific markers during the early development of human IVD [47]. Similarly, Nobuyuki Fujita et al. [46] identified CD24 as a surface marker for NPC with its high expression in the healthy and herniated NP tissue rather than in the annulus fibrosus. Furthermore, CD24 is proved to be a key marker of the irreversible cellular hierarchy during the differentiation process of the NP-progenitor cells towards NP-committed cells in mice and humans [49]. Therefore, in our study, the upregulated expression of CD24 also confirmed the positive effect of ASCs on NPC regeneration.

Functional annotation of these differentially expressed mRNAs and lncRNAs was investigated by GO and KEGG pathway analysis. The GO analysis identified significant enrichments in over 197 GO terms, including cell adhesion (GO:0007155), positive regulation of transcription from RNA polymerase II (Pol II) promoter (GO:0045944), and extracellular matrix organization (GO:0030198). These data indicate that the metabolic activities of NPCs might be enhanced by ASCs. Specifically, the cellular adhesion is crucial for stable connections between cells and tissue structure maintenance, also involved in diverse signal transduction [50]. The upregulated RNA Pol II promoter of co-cultured NPCs in the present study reflects the increased expression of protein-encoding genes. The RNA Pol II together with other factors, mediating the transcription initiation of protein-encoding genes, is an essential control point for gene expression in the eukaryotes [51]. Similarly, upregulated extracellular matrix organization would counteract the loss of extracellular matrix and further favor the regeneration of NP tissue [52].

Additionally, the most important signaling pathways altered during the co-culture were the MAPK pathway, the nuclear factor kappa-light-chain-enhancer of activated B cells (NF-kappa B) signaling pathway, and the PI3K-Akt signaling pathway (Fig. 5). Specifically, the NF-kappa B and MAPK signaling pathways, the principal regulators of inflammation and catabolism [53], are important in the symptomatic disc-degenerative diseases [54–56]. The MAPK signaling pathway was generally increased in the aged and degenerative discs [57, 58]. Our analysis displayed the downregulation of the MAPK signaling pathway, indicating degenerative NPCs could be stimulated towards normal NPCs by ASCs. In addition, NF-kappa B targets several pro-inflammatory cytokines [59–61] that are highly expressed in degenerative discs rather than normal discs. Therefore, the downregulated NF-kappa B signaling pathway of co-cultured NPCs in our study may indicate that ASCs protect NPCs from inflammatory response. Furthermore, the PI3K-Akt signaling pathway was identified as the most significantly upregulated pathway. Activated PI3K-Akt can protect against the disc degeneration [62, 63] with increased extracellular matrix synthesis [64], promoted cell proliferation [65], counteraction of cell apoptosis [66], and alleviated oxidative damage [67].

Moreover, the signal-net analysis displayed that glycogen synthase kinase 3 beta (*GSK3B*), which was downregulated, plays the most critical role in the network with the highest betweenness centrality. As a serine/threonine kinase, GSK3 is involved in the phosphorylation of numerous substrates, including signaling proteins, transcription factors and structural proteins [68, 69]. As a crucial mediator of PI3K-Akt, PKA, PKC, and Wnt/ß-catenin, GSK3 is proved to play an important role in chondrocyte differentiation [70–72]. Miclea et al. demonstrated that GSK3B could inhibit the chondrocyte proliferation and increase the cartilage apoptosis via activating the canonical Wnt signaling pathway in the ex vivo mouse embryos [73]. Also, Itoh et al. demonstrated that GSK3 proteins are involved in early stages of chondrocyte differentiation by driving the differentiation in a cell-autonomous manner [74]. In the present study, we also found that after co-culture, the degenerative NPCs expressed significantly higher *SOX9* and *COL1A2*, confirming the restored function of the NPCs.

Regarding the regulatory functions of lncRNAs, in the present study we investigated the co-expressional connection between mRNAs and lncRNAs and found that *XIST *acquired the greatest number of interactions (degree = 77) among all RNAs. *XIST*, a 17–20 kb RNA, binds the X chromosome [75, 76] to initiate X chromosome inactivation [77] and is required for whole-chromosome silencing [78]. Also, *XIST* provides one of a few tangible readouts for the stem cell quality [79] and also influences the pluripotent stem cell population, as proved in induced pluripotent stem cell treatments in regenerative medicine [80]. However, such significant roles have not studied in the context of disc degeneration and further investigations are warranted.

A competing endogenous RNAs network was constructed for the regulatory function of various lncRNAs and many miRNAs associated with disc degeneration. *miR-98-5p* is the most important in the network (interaction degree 57) followed by the *miR-27a-34* (interaction degree, 40) (Fig. 9). *miR-98* is significantly downregulated in the degenerated NP tissue and has been proved to promote type II collagen expression in NPCs [81]. Also, Li et al. reported that the downregulation of *miRNA-27b* would yield loss of type II collagen and lead to the development of IDD [27]. Therefore, the upregulation of these two miRNAs, favoring type II collagen synthesis of the NPCs, demonstrated the positive effect of ASCs on degenerative NPCs and might serve as potential therapeutic targets in IDD.

This study has some limitations. First, numerous RNA probes were investigated in the microarray analysis and this limited the validation of the gene chip results. Therefore, we only interpreted the results based on previous studies and our interests. Also, to avoid losing information, genes and lncRNAs were not further classified into specific subsets according to their functions and only the regulatory roles of differentially expressed RNAs of general interest were interpreted, serving as potential targets of the further in-depth investigation. Nevertheless, the results from our bioinformatics analysis only elucidate relevant relationships associated with these genes and lncRNAs; more in vitro or in vivo studies are necessary to comprehensively understand the specific involvement of the differentially expressed lncRNAs and mRNAs during the co-culturing process.

## Conclusion

To sum up, co-culturing human ASCs with degenerative NPCs restored the biological status of the degenerative NPCs. Our study identified the interplay between ASCs and degenerative NPCs during the co-culturing and provided valuable information for the development and application of gene therapy for IDD. More studies are required to explore the functions and mechanisms of the key RNAs involved in the regeneration of the human intervertebral disc tissue and further benefit the translation of gene therapy in IDD from bench to bedside.

## Additional files

Additional file 1: Schematic study design. (TIF 7017 kb)

Additional file 2: Supplementary materials and methods describing the isolation, culture of NPCs and ASCs, identification of ASCs, and co-culturing of NPCs and ASCs. (DOCX 131 kb)

Additional file 3: Purity and integrity of RNA. (DOCX 229 kb)

Additional file 4: Quality control of hybridization. (DOCX 1241 kb)

Additional file 5: Intervertebral disc degeneration related miRNAs identified in previous literature. (DOCX 51 kb)

Additional file 6: Top 10 differentially expressed lncRNAs and mRNAs. (DOCX 14 kb)

Additional file 7: Full list of differentially expressed mRNAs and lncRNAs. (XLSX 1394 kb)

Additional file 8: Top 20 most important pathways in the Path-net. (DOCX 15 kb)

Additional file 9: Top 20 most significantly regulated genes in signal-net. (DOCX 15 kb)

## Abbreviations

*ACAN*: Aggrecan
ASCs: Adipose-derived mesenchymal stem cells
ceRNA: Competing endogenous RNA
*COL2A1*: Type II collagen
*ENPP1*: Ectonucleotide pyrophosphatase 1
FDR: False discovery rate
GEO: Gene Expression Omnibus
GO: Gene Ontology
*GSK3B*: Glycogen synthase kinase 3 beta
IDD: Intervertebral disc degeneration
*ITGA*: Integrin alpha
KEGG: Kyoto Encyclopedia of Genes and Genomes
lncRNAs: Long non-coding RNAs
*MAPK*: Mitogen-activated protein kinase
miRNAs: Micro RNAs
MREs: Micro RNA response elements
mRNA: Messenger RNA
MSCs: Mesenchymal stem cells
*MT1F*: Metallothionein 1F
*NF-kappa B*: Kappa-light-chain-enhancer of activated B cells
NP: Nucleus pulposus
NPCs: Nucleus pulposus cells
*OPN*: Osteopontin
*PI3K*: Phosphoinositide 3-kinase
*PIK3CB*: Phosphoinositide-3-kinase, catalytic, beta polypeptide
*PIK3R3*: Phosphoinositide-3-kinase, regulatory subunit 3
*Pol II*: Polymerase II
RVM: Random variance model
*SOX9*: Sex determining region Y box 9
*SPP1*: Secreted phosphoprotein 1
*XIST*: X-inactive specific transcript
