# Supplementary figures and images for: Aberrantly expressed messenger RNAs and long noncoding RNAs in degenerative nucleus pulposus cells co-cultured with adipose-derived mesenchymal stem cells
Source: Arthritis Res Ther. 2018 Aug 16;20:182. doi: 10.1186/s13075-018-1677-x (PMC6097446; doi:10.1186/s13075-018-1677-x)

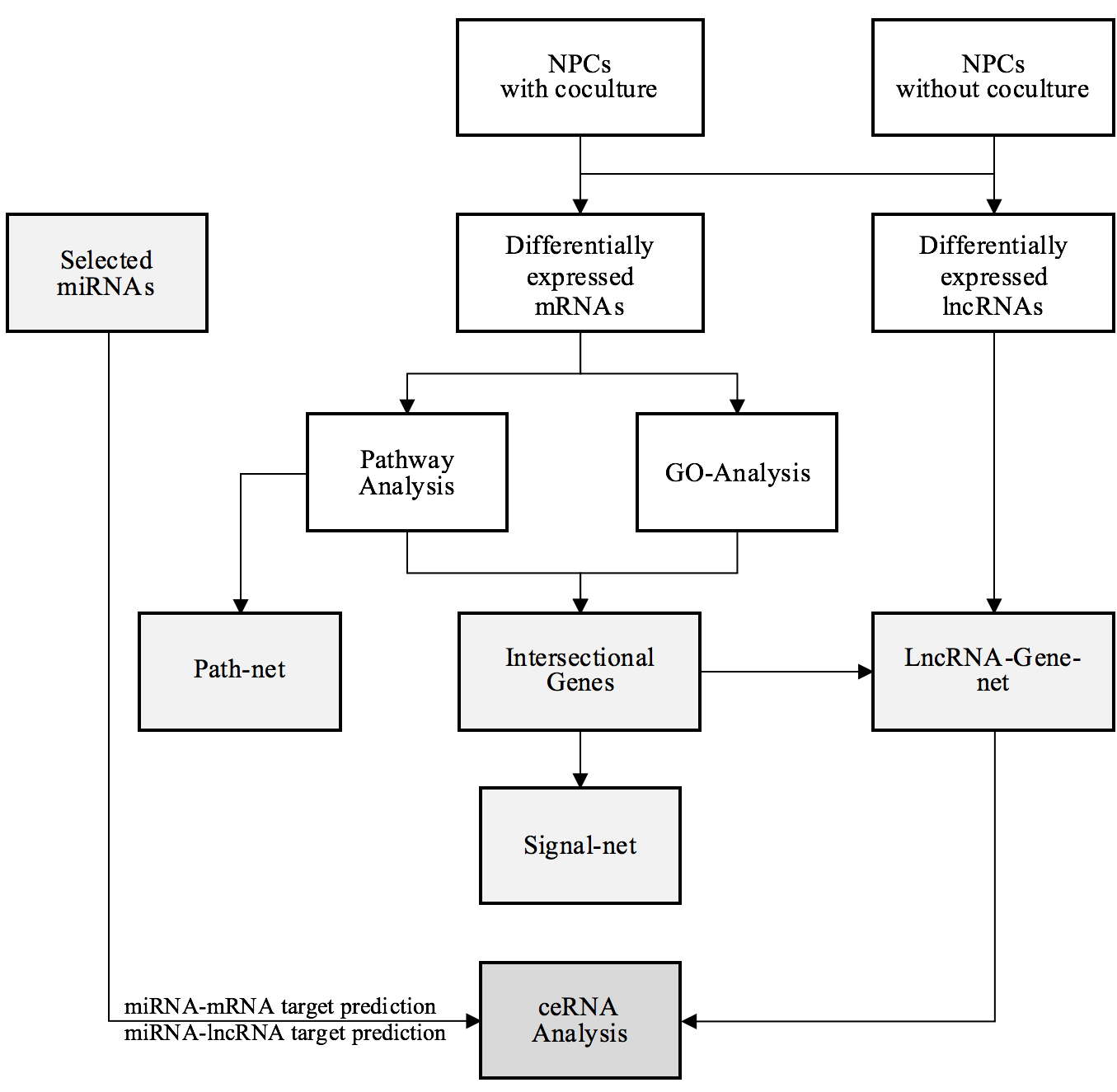

Supplement: Supplementary file 1 — Schematic study design. (TIF 7017 kb) [file 13075_2018_1677_MOESM1_ESM.tif]
