# Supplementary material for: Aberrantly expressed messenger RNAs and long noncoding RNAs in degenerative nucleus pulposus cells co-cultured with adipose-derived mesenchymal stem cells
Source: Arthritis Res Ther. 2018 Aug 16;20:182. doi: 10.1186/s13075-018-1677-x (PMC6097446; doi:10.1186/s13075-018-1677-x)
Supplement: Supplementary file 2 — Supplementary materials and methods describing the isolation, culture of NPCs and ASCs, identification of ASCs, and co-culturing of NPCs and ASCs. (DOCX 131 kb) [file 13075_2018_1677_MOESM2_ESM.docx]

**Additional file 2**

**Isolation and culture of NP derived cells (NPCs)**

The isolation of the NPCs was performed as previously described[1, 2]. Briefly, the NP tissue was carefully washed in phosphate buffered saline (PBS), blood clots and annulus fibrous tissue were cautiously removed. Then the NP tissue was transferred into serum-free medium supplemented with 1% penicillin/streptomycin, and cut into small pieces while treated with 0.4 mg/mL collagenase II (Sigma, St Louis, USA) at 37° for 5 hours. After digestion, the cells were collected firstly by passing through the 100 μm cell-strainer to remove the tissue debris, then by centrifugation at 400g for 5min to pellet the cells. Subsequently, the cells were re-suspended after removal of the supernatant and cultured in Dulbecco’s modified Eagle’s medium/F12 medium (DMEM/F12, Hyclone, South Logan, USA), containing 10% fetal bovine serum (FBS) in 37°, 5% CO2 incubator. The medium was changed every 2-3 days.

**Isolation and culture of adipose-derived stem cells (ASCs)**

The adipose tissue was obtained from patients’ back during the spinal operation and ASCs isolation followed the previously reported method [3, 4]. Briefly, the adipose tissue was harvested and stored in DMEM, then transferred to the lab. After washed twice with PBS to remove blood, fat tissue was minced into 1mm^2^ small pieces, subsequently was treated with 0.1% collagenase type I (Sigma, St Louis, USA) in 37° for 60min with permanent shaking. The cell-tissue mixture thereafter was flirted by 100μm nylon mesh, then centrifuged and the supernatant was discarded. The pallet was re-suspended using DMEM/F12 (Hyclone, South Logan, USA) containing 10% FBS and 1% penicillin/streptomycin in 37°, 5% CO2 incubator. The medium was changed every 2-3 days and the cells were passaged when the confluence achieved 80%.

**Identification of ASCs**

To demonstrate the stemness of ASCs, the flow cytometry test was performed to identify the positive and negative markers related to ASCs. When isolated cells of the initial passage were cultured to reach 80% confluence, they were used for cell marker analysis. The cells were incubated with the phycoerythrin (PE)-conjugated antibodies (all from eBioscience, CA, USA): CD105, CD90, CD34, CD45, human leukocyte antigen(HLA)-DR, HLA-ABC. Target cells with positive CD105, CD90, and negative CD34, CD45 were selected for further study. Furthermore, the ASCs were induced to multilineage differentiate using specific media as described before[5], in vitro differentiation of ASCs was proved by histological assays: Oil Red O (adipogenesis), Alizarin Red and Alkaline Phosphatase (ALP) (osteogenesis) and Alcian blue (chondrogenesis), respectively.

**Reference:**

1. Ruan D, Zhang Y, Wang D, Zhang C, Wu J, Wang C, Shi Z, Xin H, Xu C, Li H *et al*: Differentiation of human Wharton's jelly cells toward nucleus pulposus-like cells after coculture with nucleus pulposus cells in vitro. *Tissue Eng Part A* 2012, 18(1-2):167-175.

2. Han Z, Zhang Y, Gao L, Jiang S, Ruan D: Human Wharton's Jelly Cells Activate Degenerative Nucleus Pulposus Cells In Vitro. *Tissue Eng Part A* 2018, doi: 10.1089/ten.TEA.2017.0340.

3. Russo V, Yu C, Belliveau P, Hamilton A, Flynn LE: Comparison of human adipose-derived stem cells isolated from subcutaneous, omental, and intrathoracic adipose tissue depots for regenerative applications. *Stem Cells Transl Med* 2014, 3(2):206-217.

4. Schaffler A, Buchler C: Concise review: adipose tissue-derived stromal cells--basic and clinical implications for novel cell-based therapies. *Stem Cells* 2007, 25(4):818-827.

5. Zuk PA, Zhu M, Ashjian P, De Ugarte DA, Huang JI, Mizuno H, Alfonso ZC, Fraser JK, Benhaim P, Hedrick MH: Human adipose tissue is a source of multipotent stem cells. *Mol Biol Cell* 2002, 13(12):4279-4295.
