# Supplementary material for: Aberrantly expressed messenger RNAs and long noncoding RNAs in degenerative nucleus pulposus cells co-cultured with adipose-derived mesenchymal stem cells
Source: Arthritis Res Ther. 2018 Aug 16;20:182. doi: 10.1186/s13075-018-1677-x (PMC6097446; doi:10.1186/s13075-018-1677-x)
Supplement: Supplementary file 3 — Purity and integrity of RNA. (DOCX 229 kb) [file 13075_2018_1677_MOESM3_ESM.docx]

| Sample | Concentration (ng/μL) | 260 | 280 | Ratio (260/280) | 260 Raw | 280 Raw | 320 Raw |
| --- | --- | --- | --- | --- | --- | --- | --- |
| C1 | 420.46 | 0.526 | 0.275 | 1.908 | 0.571 | 0.335 | 0.074 |
| C2 | 729.035 | 0.911 | 0.472 | 1.931 | 0.91 | 0.499 | 0.058 |
| C3 | 604.231 | 0.755 | 0.401 | 1.886 | 0.769 | 0.435 | 0.058 |
| E1 | 562.479 | 0.703 | 0.367 | 1.918 | 0.725 | 0.409 | 0.065 |
| E2 | 710.773 | 0.888 | 0.46 | 1.933 | 0.894 | 0.492 | 0.061 |
| E3 | 782.464 | 0.978 | 0.507 | 1.929 | 0.975 | 0.536 | 0.063 |

**Additional file 3: Purity and integrity of RNA**

**Additional table 1:** Purity of RNA by spectrophotometric analysis


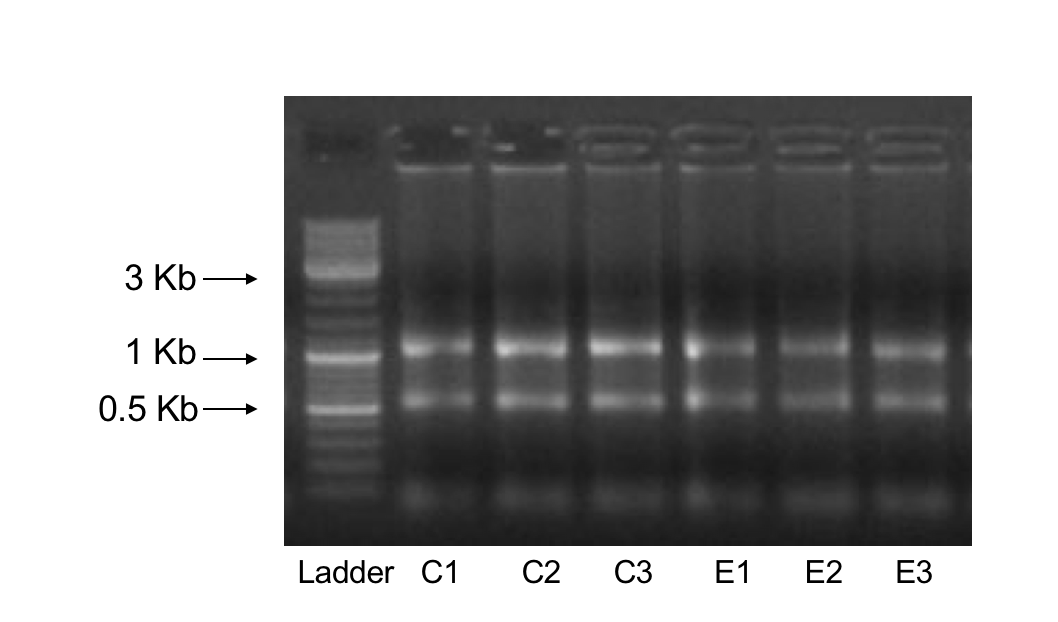


**Additional figure 1.** Integrity of RNA by agarose gel electrophoresis. As shown on the figure that each RNA sample had two distinct bands at 28s and 18s showing the integrity of the RNA sample.
