# Supplementary material for: Aberrantly expressed messenger RNAs and long noncoding RNAs in degenerative nucleus pulposus cells co-cultured with adipose-derived mesenchymal stem cells
Source: Arthritis Res Ther. 2018 Aug 16;20:182. doi: 10.1186/s13075-018-1677-x (PMC6097446; doi:10.1186/s13075-018-1677-x)
Supplement: Supplementary file 4 — Quality control of hybridization. (DOCX 1241 kb) [file 13075_2018_1677_MOESM4_ESM.docx]

**Additional file 4: Quality control of hybridization (Affymetrix Gene 2.0)**


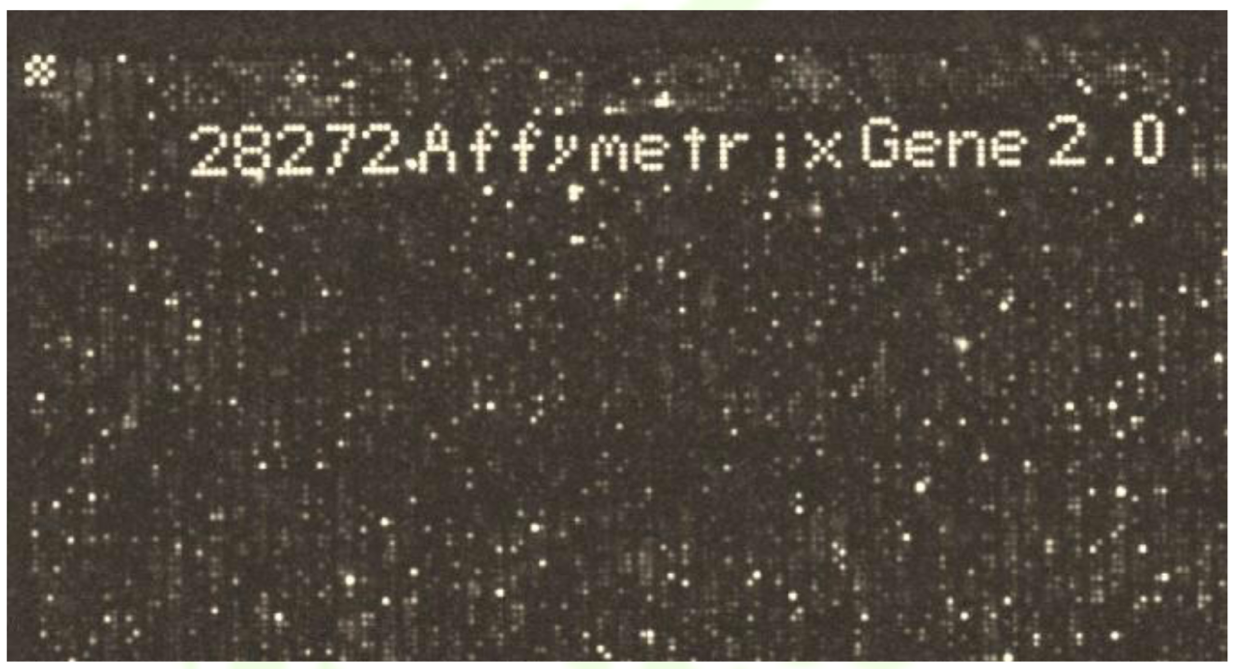


**Additional figure 2.** Control Oligo B2. As pictured that Oligo B2 hybridization was strongly positive, showing chip array name “ 28272. AffymetrixGene2.0” and the checkerboard pattern at the upper left corner, this indicate the high quality of the microarray chip.


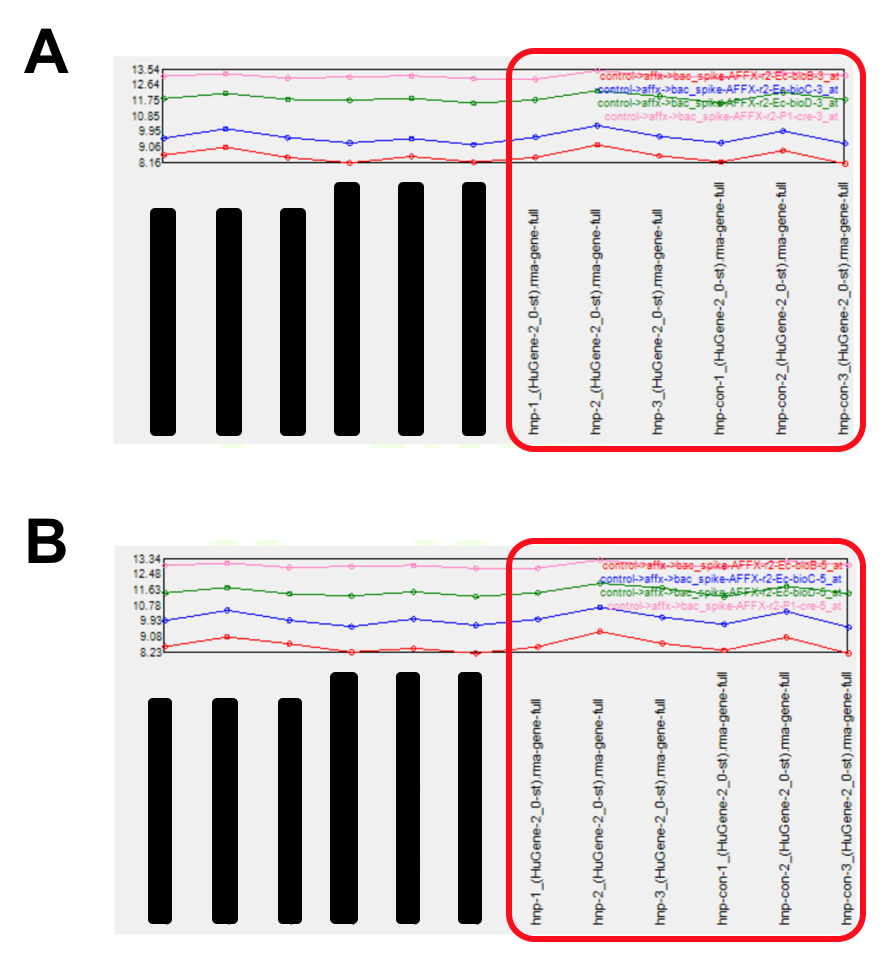


**Additional figure 3.** Hybridization Controls. Through (**A**) 3’ Hybridization and (**B**) 5’ Hybridization following the manufacturer’s instruction, we found in both hybridization that BioB < BioC < BioD < Cre, suggesting good quality of the gene chip. hnp: human NP cells after coculturing with ASCs; hnp-con: human NP cells without coculturing.
