# Supplementary material for: Aberrantly expressed messenger RNAs and long noncoding RNAs in degenerative nucleus pulposus cells co-cultured with adipose-derived mesenchymal stem cells
Source: Arthritis Res Ther. 2018 Aug 16;20:182. doi: 10.1186/s13075-018-1677-x (PMC6097446; doi:10.1186/s13075-018-1677-x)
Supplement: Supplementary file 5 — Intervertebral disc degeneration related miRNAs identified in previous literature. (DOCX 51 kb) [file 13075_2018_1677_MOESM5_ESM.docx]

**Additional file 5: Intervertebral disc degeneration related miRNAs from previous literatures**

| **miRNA** | **Literature** |
| --- | --- |
| hsa-miR-98-5p | [1, 2] |
| hsa-miR-27a-3p | [3, 4] |
| hsa-miR-146a-3p | [5] |
| hsa-miR-146a-5p | [6] |
| hsa-miR-93-5p | [7] |
| hsa-miR-494-3p | [8, 9] |
| hsa-miR-21-3p | [10-12] |
| hsa-miR-10b-5p | [13] |
| hsa-miR-10b-3p | [13] |
| hsa-miR-27a-5p | [3] |
| hsa-miR-100-3p | [14] |
| hsa-miR-27b-3p | [15] |
| hsa-miR-93-3p | [7] |
| hsa-miR-494-5p | [8, 9] |
| hsa-miR-21-5p | [10-12] |
| hsa-miR-140-5p | [5] |
| hsa-miR-27b-5p | [15] |
| hsa-miR-98-3p | [1] |
| hsa-miR-193a-3p | [16] |
| hsa-miR-155-5p | [17] |
| hsa-miR-140-3p | [5] |
| hsa-miR-193b-3p | [18] |
| hsa-miR-133a-3p | [19] |

**Reference:**

1. Ji ML, Lu J, Shi PL, Zhang XJ, Wang SZ, Chang Q, Chen H, Wang C. Dysregulated miR-98 Contributes to Extracellular Matrix Degradation by Targeting IL-6/STAT3 Signaling Pathway in Human Intervertebral Disc Degeneration. *J Bone Miner Res* 2016, 31(4):900-909.

2. Tan B, Wang H, Dong J, Yuan Z, Wang D, Wang F. Dysregulated miR-98-5p contributes to excessive apoptosis of nucleus pulposus cells by BMP2 in human intervertebral disc degeneration. *Biomed Res* 2017, 28(9).

3. Liu G, Cao P, Chen H, Yuan W, Wang J, Tang X. MiR-27a regulates apoptosis in nucleus pulposus cells by targeting PI3K. *PloS one* 2013, 8(9):e75251.

4. Cao Z, Chen L. Inhibition of miR-27a suppresses the inflammatory response via the p38/MAPK pathway in intervertebral disc cells. *Exp Ther Med* 2017, 14(5):4572-4578.

5. Zhao B, Yu Q, Li H, Guo X, He X. Characterization of microRNA expression profiles in patients with intervertebral disc degeneration. *Int J Mol Med* 2014, 33(1):43-50.

6. Xi Y, Wu X, Wang Y, Xu N, Zhang X. MicroRNA-146a-5p inhibits recruitment of macrophages and protects nucleus pulposus cells from TNF-α-induced apoptosis by targeting TRAF6. *Int J Clin Exp Pathol* 2016, 9(3):3267-3276.

7. Jing W, Jiang W. MicroRNA-93 regulates collagen loss by targeting MMP3 in human nucleus pulposus cells. *Cell Prolif* 2015, 48(3):284-292.

8. Kang L, Yang C, Song Y, Zhao K, Liu W, Hua W, Wang K, Tu J, Li S, Yin H *et al*. MicroRNA-494 promotes apoptosis and extracellular matrix degradation in degenerative human nucleus pulposus cells. *Oncotarget* 2017, 8(17):27868-27881.

9. Wang T, Li P, Ma X, Tian P, Han C, Zang J, Kong J, Yan H. MicroRNA-494 inhibition protects nucleus pulposus cells from TNF-alpha-induced apoptosis by targeting JunD. *Biochimie* 2015, 115:1-7.

10. Lin H, Zhang W, Zhou T, Li W, Chen Z, Ji C, Zhang C, He F: Mechanism of microRNA-21 regulating IL-6 inflammatory response and cell autophagy in intervertebral disc degeneration. *Exp Ther Med* 2017, 14(2):1441-1444.

11. Chen B, Huang SG, Ju L, Li M, Nie FF, Zhang Y, Zhang YH, Chen X, Gao F. Effect of microRNA-21 on the proliferation of human degenerated nucleus pulposus by targeting programmed cell death 4. *Rev Bras Ginecol Obstet* 2016, 49(6).

12. Liu H, Huang X, Liu X, Xiao S, Zhang Y, Xiang T, Shen X, Wang G, Sheng B. miR-21 promotes human nucleus pulposus cell proliferation through PTEN/AKT signaling. *Int J Mol Sci* 2014, 15(3):4007-4018.

13. Yu X, Li Z, Shen J, Wu WK, Liang J, Weng X, Qiu G. MicroRNA-10b promotes nucleus pulposus cell proliferation through RhoC-Akt pathway by targeting HOXD10 in intervetebral disc degeneration. *PloS one* 2013, 8(12):e83080.

14. Yan N, Yu S, Zhang H, Hou T. Lumbar Disc Degeneration is Facilitated by MiR-100-Mediated FGFR3 Suppression. *Cell Physiol Biochem* 2015, 36(6):2229-2236.

15. Li HR, Cui Q, Dong ZY, Zhang JH, Li HQ, Zhao L. Downregulation of miR-27b is Involved in Loss of Type II Collagen by Directly Targeting Matrix Metalloproteinase 13 (MMP13) in Human Intervertebral Disc Degeneration. *Spine* 2016, 41(3):E116-123.

16. Ji ML, Zhang XJ, Shi PL, Lu J, Wang SZ, Chang Q, Chen H, Wang C. Downregulation of microRNA-193a-3p is involved in invertebral disc degeneration by targeting MMP14. *J Mol Med (Berl)* 2016, 94(4):457-468.

17. Wang HQ, Yu XD, Liu ZH, Cheng X, Samartzis D, Jia LT, Wu SX, Huang J, Chen J, Luo ZJ. Deregulated miR-155 promotes Fas-mediated apoptosis in human intervertebral disc degeneration by targeting FADD and caspase-3. *J Pathol* 2011, 225(2):232-242.

18. Ukai T, Sato M, Akutsu H, Umezawa A, Mochida J. MicroRNA-199a-3p, microRNA-193b, and microRNA-320c are correlated to aging and regulate human cartilage metabolism. *J Orthop Res* 2012, 30(12):1915-1922.

19. Xu YQ, Zhang ZH, Zheng YF, Feng SQ. Dysregulated miR-133a Mediates Loss of Type II Collagen by Directly Targeting Matrix Metalloproteinase 9 (MMP9) in Human Intervertebral Disc Degeneration. *Spine* 2016, 41(12):E717-724.
