# Supplementary material for: Aberrantly expressed messenger RNAs and long noncoding RNAs in degenerative nucleus pulposus cells co-cultured with adipose-derived mesenchymal stem cells
Source: Arthritis Res Ther. 2018 Aug 16;20:182. doi: 10.1186/s13075-018-1677-x (PMC6097446; doi:10.1186/s13075-018-1677-x)
Supplement: Supplementary file 6 — Top 10 differentially expressed lncRNAs and mRNAs. (DOCX 14 kb) [file 13075_2018_1677_MOESM6_ESM.docx]

**Additional file 6: Top 10 differentially expressed lncRNAs and mRNAs**

| **Top 10 differentially expressed lncRNAs** | | | |
| --- | --- | --- | --- |
| **Gene Symbol** | **Style** | **Fold-change** | **P-value** |
| C3orf49 | up | 18.9 | < 1E-07 |
| LOC440895 | up | 9.1 | < 1E-07 |
| TTTY15 | up | 5.9 | 1.0E-7 |
| SNORA14A | up | 5.6 | < 1e-07 |
| MT1L | up | 4.2 | 7.68E-5 |
| ECRP | up | 4.0 | 2.0E-7 |
| SCARNA8 | up | 3.8 | 1.0E-7 |
| FAM7A3 | up | 3.4 | 3.0E-7 |
| FAM7A2 | up | 3.3 | 3.0E-7 |
| SNORD116-1 | up | 3.0 | 1.136E-4 |
| **Top 10 differentially expressed mRNAs** | | | |
| SPP1 | up | 106.4 | < 1E-07 |
| MT1F | up | 76.9 | < 1E-07 |
| ENPP1 | up | 34.5 | < 1E-07 |
| EPYC | up | 34.5 | < 1E-07 |
| CD24 | up | 20.4 | < 1E-07 |
| C4orf31 | up | 17.9 | < 1E-07 |
| ZNF385B | up | 16.7 | < 1E-07 |
| KDR | up | 13.5 | < 1E-07 |
| EHD3 | up | 12.5 | < 1E-07 |
| GAS7 | up | 12.5 | < 1E-07 |
