# Supplementary material for: Aberrantly expressed messenger RNAs and long noncoding RNAs in degenerative nucleus pulposus cells co-cultured with adipose-derived mesenchymal stem cells
Source: Arthritis Res Ther. 2018 Aug 16;20:182. doi: 10.1186/s13075-018-1677-x (PMC6097446; doi:10.1186/s13075-018-1677-x)
Supplement: Supplementary file 8 — Top 20 most important pathways in the Path-net. (DOCX 15 kb) [file 13075_2018_1677_MOESM8_ESM.docx]

**Additional file 8: Top 20 most important pathways in the Path-net**

| **Path name** | **Style** | **In-degree** | **Out-degree** | **Degree** |
| --- | --- | --- | --- | --- |
| MAPK signaling pathway | all* | 30 | 5 | 35 |
| Apoptosis | down **^†^** | 22 | 3 | 25 |
| Pathways in cancer | all | 0 | 21 | 21 |
| Cell cycle | down | 15 | 3 | 18 |
| Focal adhesion | all | 7 | 8 | 15 |
| Wnt signaling pathway | down | 6 | 8 | 14 |
| Calcium signaling pathway | down | 9 | 4 | 13 |
| Cytokine-cytokine receptor interaction | all | 13 | 0 | 13 |
| Glycolysis / Gluconeogenesis | down | 9 | 3 | 12 |
| p53 signaling pathway | down | 10 | 2 | 12 |
| Adherens junction | down | 6 | 5 | 11 |
| Jak-STAT signaling pathway | down | 6 | 5 | 11 |
| TGF-beta signaling pathway | down | 6 | 4 | 10 |
| VEGF signaling pathway | down | 4 | 5 | 9 |
| Regulation of actin cytoskeleton | all | 6 | 3 | 9 |
| T cell receptor signaling pathway | down | 3 | 5 | 8 |
| Pancreatic cancer | up**^‡^** | 1 | 7 | 8 |
| Melanoma | up | 2 | 6 | 8 |
| Ubiquitin mediated proteolysis | down | 8 | 0 | 8 |
| Phosphatidylinositol signaling system | down | 6 | 1 | 7 |

* all: both down- and up-regulated; † down: down-regulated; ‡ up: up-regulated
