# Supplementary material for: Aberrantly expressed messenger RNAs and long noncoding RNAs in degenerative nucleus pulposus cells co-cultured with adipose-derived mesenchymal stem cells
Source: Arthritis Res Ther. 2018 Aug 16;20:182. doi: 10.1186/s13075-018-1677-x (PMC6097446; doi:10.1186/s13075-018-1677-x)
Supplement: Supplementary file 9 — Top 20 most significantly regulated genes in signal-net. (DOCX 15 kb) [file 13075_2018_1677_MOESM9_ESM.docx]

**Additional file 9: Top 20 most significantly regulated genes in signal-net**

| **Gene**  **symbol** | **Description** | **Style** | **In-degree** | **Out-degree** | **Degree** |
| --- | --- | --- | --- | --- | --- |
| PIK3R3 | phosphoinositide-3-kinase, regulatory subunit 3 (gamma) | up | 17 | 6 | 19 |
| PIK3CB | phosphoinositide-3-kinase, catalytic, beta polypeptide | down | 16 | 5 | 18 |
| FGFR2 | fibroblast growth factor receptor 2 | up | 11 | 4 | 15 |
| MET | met proto-oncogene (hepatocyte growth factor receptor) | down | 10 | 4 | 14 |
| MAPK14 | mitogen-activated protein kinase 14 | down | 6 | 8 | 14 |
| PLCB4 | phospholipase C, beta 4 | down | 13 | 9 | 13 |
| KDR | kinase insert domain receptor (a type III receptor tyrosine kinase) | up | 10 | 3 | 13 |
| KIT | v-kit Hardy-Zuckerman 4 feline sarcoma viral oncogene homolog | down | 10 | 3 | 13 |
| GNB4 | guanine nucleotide binding protein (G protein), beta polypeptide 4 | down | 2 | 10 | 12 |
| FOS | FBJ murine osteosarcoma viral oncogene homolog | down | 6 | 6 | 11 |
| JAK2 | Janus kinase 2 | down | 7 | 4 | 11 |
| JUN | jun proto-oncogene | down | 5 | 8 | 11 |
| ITGA6 | integrin, alpha 6 | up | 11 | 1 | 11 |
| ITGA1 | integrin, alpha 1 | up | 11 | 1 | 11 |
| ITGA3 | integrin, alpha 3 (antigen CD49C, alpha 3 subunit of VLA-3 receptor) | up | 11 | 1 | 11 |
| ITGA4 | integrin, alpha 4 (antigen CD49D, alpha 4 subunit of VLA-4 receptor) | down | 11 | 1 | 11 |
| ITGA7 | integrin, alpha 7 | down | 11 | 1 | 11 |
| ITGA10 | integrin, alpha 10 | up | 11 | 1 | 11 |
| ITGA8 | integrin, alpha 8 | down | 11 | 1 | 11 |
| SOCS1 | suppressor of cytokine signaling 1 | up | 2 | 8 | 10 |
